# Quality of nursing records in cardiac arrest based on the In-hospital Utstein Style protocol

**DOI:** 10.1590/0034-7167-2024-0572

**Published:** 2026-08-03

**Authors:** Dhieizom Rodrigo de Souza, Lucia Tobase, Débora Rodrigues Vaz, Thatiane Facholi Polastri, Sandra Helena Cardoso, Heloisa Helena Cueto Peres

**Affiliations:** I Universidade de São Paulo. São Paulo, São Paulo, Brazil; II Centro Universitário São Camilo. São Paulo, São Paulo, Brazil

**Keywords:** Nursing Records, Nursing Care, Heart Arrest, Patient Safety, Nursing., Registros de Enfermería, Atención de Enfermería, Paro Cardíaco, Seguridad del Paciente, Enfermería.

## Abstract

**Objectives:**

to assess the quality of nursing records in cardiac arrest based on the In-hospital Utstein Style.

**Methods:**

a quantitative, quasi-experimental, non-randomized, before-and-after study, with intervention using a printed form to support nursing documentation in cardiac arrest.

**Results:**

overall compliance for completion and completeness was assessed for 89 records using a data collection form developed from In-hospital Utstein Style variables. The overall compliance of nursing records showed an increase in the mean percentage of completion, from 55.57% to 65.25% (p=0.003), and in completeness, from 68.66% to 76.54% (p=0.001), comparing the preand post-intervention periods.

**Conclusions:**

the intervention showed a statistically significant effect on the overall compliance rates for completion and completeness of nursing records in cardiac arrest. The study contributed to promoting the importance of using protocols as an essential element in recording care during cardiac arrest and in the implementation of continuous training for the nursing staff.

## RESUMEN

**Objetivos:**

evaluar la calidad de registros de enfermería en casos de paro cardiorrespiratorio según In-hospital Utstein Style.

**Métodos:**

estudio cuantitativo, cuasiexperimental, no aleatorizado, antes-después, con la intervención de formulario de apoyo a registros de enfermería en casos de paro cardiorrespiratorio.

**Resultados:**

se evaluó el cumplimiento general en cuanto al llenado y la integridad de 89 registros mediante formulario de recolección de datos desarrollado usando variables del In-hospital Utstein Style. El cumplimiento general de los registros de enfermería mostró un aumento en el porcentaje promedio de llenado (55,57%-65,25% (p=0,003)) y la integridad (68,66%-76,54% (p=0,001)), al comparar los períodos previos y posteriores a la intervención.

**Conclusiones:**

la intervención mostró un efecto estadísticamente significativo en tasas generales de cumplimiento en el llenado y cumplimentación de los registros de enfermería en casos de paro cardiorrespiratorio. El estudio contribuyó a resaltar la importancia del uso de protocolos como elemento esencial en el registro de atención durante el paro cardiorrespiratorio y en la implementación de formación continua del equipo de enfermería.

## INTRODUCTION

The epidemiology of in-hospital cardiac arrest (CA) is still limited by a scarcity of global and regional data, especially in lowand middle-income countries^(1)^. To improve care and assess effectiveness in post-CA survival, specific guidelines and protocols for recording the event have emerged^(2)^.

The Utstein Style and In-hospital Utstein Style protocols, developed in the 1990s, offer standardized guidelines for documenting out-of-hospital and in-hospital CA, respectively^(2)^. These records allow for comparison of the epidemiological profile of CA between different services and countries, organizing information from the moment of CA to the outcomes, with patient follow-up up to one year after discharge^(3-5)^.

In Brazil, the In-hospital Utstein Style has been translated, adapted, and updated to improve data collection and balance standardization with clinical practice and care outcomes^(4)^. Nursing plays an essential role in record keeping, which is not only a legal obligation but also a crucial tool for audits, teaching, and research, promoting scientific advancement and continuous improvement of care^(2,6,7)^.

However, the lack of complete information during care hinders the assessment of resuscitation quality and the development of care indicators^(7)^. As part of a multidisciplinary staff, nursing staff must ensure safe and accurate records, aligned with safety goals and the Brazilian National Patient Safety Program^(8-11)^.

Although studies have assessed the quality of nursing records, few focus specifically on compliance of notes related to CA^(2,3,11)^. In this study, compliance was assessed by considering the completeness of In-hospital Utstein Style items in CA notes made by nursing staff. The absence of one or more standardized elements was understood as non-compliance^(12)^.

Investigating the quality of these records is essential to strengthen patient safety and ensure practice grounded in ethical and legal principles. The study hypothesis is that, after an educational intervention, nursing records in CA show greater conformity in terms of completion and accuracy.

### Study relevance

This article is derived from Dissertation 003219693^(13)^, deposited in the *Universidade de São Paulo*’s Institutional Repository, available at: https://doi.org/10.11606/D.7.2022.tde-30102024-172947.

## OBJECTIVES

To assess the quality of nursing records in CA based on the In-hospital Utstein Style.

## METHODS

### Ethical aspects

The research was approved under the Ethical Opinion according to Resolution 466/2012 of the Brazilian National Health Council. The study was conducted in accordance with national and international ethical guidelines, and was approved by the Research Ethics Committee of the School of Nursing at the *Universidade de São Paulo*. Informed Consent Form was obtained from all individuals involved in the study.

### Study design, period, and location

This is a quantitative, quasi-experimental, before-and-after study conducted between January and July 2021. The study site was a university hospital in São Paulo, with data collection in the Adult Intensive Care Unit (AICU) and the Adult Emergency Room (AER), due to patents’ severity and higher incidence of CA. The study methodology was guided by the Standards for Quality Improvement Reporting Excellence guidelines from the Equator Network platform.

### Sample and inclusion criteria

The study sample consisted of 89 CA records, collected at each phase of the research. The sample size calculation, based on 20 initial records, determined a sample size capable of detecting a 55% difference in the compliance rate with a statistical power of 80%. The study included medical records of patients who experienced a CA during their last hospitalization and who had complete and legible notes about the event.

### Data collection

Prior to the intervention, data were collected retrospectively from the medical records of patients who suffered CA between July 2020 and February 2021, always in a private setting to ensure confidentiality. Only nursing records were analyzed, focusing on nursing notes and progress notes. The data collection form was developed based on the In-hospital Utstein Style protocol, containing 14 variables grouped into three domains:

- Pre-event (two items): monitoring and immediate cause.
- CA process (six items): date and time of event, witness present, intervention of Advanced Cardiovascular Life Support (ACLS), initial rhythm, cardiopulmonary resuscitation (CPR) attempt, and treatments during the event.
- Results (one item): event outcome at the hospital.

In addition, supplementary variables were included, such as initial clinical condition, timing of events, need for intubation, CA provider, and legibility of notes.

### Intervention and training

Training took place between February 1 and 5, 2021, with 132 nursing professionals (60 from the AICU and 72 from the AER), including eight residents. Training addressed the importance of CA documentation based on the In-hospital Utstein Style protocol, focusing on data completeness.

For this purpose, a three-minute microlearning module was used, with an interactive video and avatars explaining basic and ethical concepts, narrated by the researcher. After the video, the researcher demonstrated how to fill out the form and answered questions. Each training session lasted 15 minutes.

### Post-intervention phase

This phase took place in the first half of 2021, and the data were collected from medical records to compare the quality of records after training.

### Data treatment

**Figure 1:**
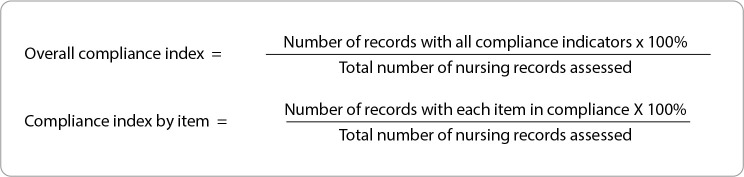
Formulas for overall compliance index and compliance index per item

Data analysis was performed using descriptive statistics to characterize the sample and inferential statistics to test the hypotheses. The non-parametric Wilcoxon-Mann-Whitney test was used to compare numerical variables. For categorical variables, Pearson’s chi-squared test was adopted. The significance level for all tests was 5%. Record compliance was calculated as the ratio between the number of complete records and the total number of records assessed, as shown in Figure 1.

## RESULTS

A total of 89 nursing records in CA were analyzed, before and after the intervention, and it was found that, respectively, 63 (70.79%)/67 (75.28%) records were in the AER and 26 (29.21%)/22 (24.72%) records were in the AICU.

An increase was observed in the mean compliance of nursing record completion in CA, when compared with the In-hospital Utstein Style protocol, in the pre-intervention (55.57%) and post-intervention (65.25%) periods, p=0.003 in the AICU and AER. Table 1 presents the variables that obtained significant compliance rates regarding completion in the preand post-intervention periods.

**Table 1:** Presentation of compliance and non-compliance regarding the completion of each variable in the nursing record of cardiac arrest in the preand post-intervention moments of the In-hospital Utstein Style protocol, São Paulo, São Paulo, Brazil, 2021

Variable	According to Non-compliant	Moment				*p* value^†^
		Pre		Post		
		N	%	N	(%)	
Monitored	Compliant	40	44.94	58	65.17	0.007
	Non-compliant	49	55.06	31	34.83	
ACLS/CA interventions	Compliant	27	30.34	45	50.56	0.006
	Non-compliant	62	69.66	44	49.44	
OTI schedule	Compliant	48	53.93	66	74.16	0.005
	Non-compliant	41	46.07	23	25.84	
Treatment during the event	Compliant	30	33.71	49	55.06	0.004
	Non-compliant	59	66.29	40	44.94	
CA provider	Compliant	17	19.10	37	41.57	0.001
	Non-compliant	72	80.90	52	58.43	

*CA - cardiac arrest; †p value - Pearson’s chi-squared test; ACLS - advanced cardiovascular life support; OTI - orotracheal intubation.*

In other variables, no statistical significance was observed, despite the differentiation of compliance values in records at the preand post-intervention moments, such as: date of CA (83 (93.26%)/88 (98.88%) (p=0.055)); witnessed CA (55 (61.68%)/64 (71.91%) (p=0.153); immediate cause of CA (35 (39.33%)/40 (44.94%) (p=0.449)); attempted resuscitation (56 (62.92%)/62 (69.66%) (p=0.343)); patient initial condition (38 (42.70%)/40 (44.94%) (p=0.763)); initial rhythm in CA (43 (48.31%)/35 (39.33%) (p=0.701)); time of event (85 (95.51%)/86 (96.63%) (p=0.701)); and event outcome (86 (96.63%)/85 (95.51%) (p=0.701)).

Furthermore, an increase was observed in the mean completeness of the information documented in nursing records, from 68.66% to 76.54% (p=0.001), before and after the implementation of the support form in the AICU and AER.

Table 2 presents the variables that obtained significant compliance rates regarding completeness in the preand post-intervention periods.

**Table 2:** Distribution of relative completeness regarding data entry according to the variable in the nursing record of cardiac arrest in the preand post-intervention moments of the In-hospital Utstein Style pilot protocol, São Paulo, São Paulo, Brazil, 2021

Variable	Degree of completeness	Moment				*p* value^†^
		Pre		Post		
		N	%	N	(%)	
Event schedule	Complete	15	16.85	27	30.34	0.020
	Partially complete	34	38.20	19	21.35	
	Incomplete	36	40.45	40	44.94	
	Not applicable	4	4.49	3	3.37	
Treatment during the event	Complete	25	28.09	47	52.81	0.012
	Partially complete	2	2.25	1	1.12	
	Incomplete	3	3.37	1	1.12	
	Not applicable	59	66.29	40	44.94	
Event result	Complete	50	56.18	78	87.64	<0.001
	Partially complete	30	33.71	2	2.25	
	Incomplete	6	6.74	5	5.62	
	Not applicable	3	3.37	4	4.49	

*†p value - Pearson’s chi-square test.*

In other variables, no statistical significance was observed in the results, despite showing differences in the completeness values of records in the preand post-intervention periods, such as: data (71 (79.78%)/75 (84.27%) (p=0.498)); witnessed (52 (58.43%)/61 (68.54%) (p=0.712)); monitored (36 (40.45%)/54 (60.67%) (p=0.144)); advanced support interventions at the time of CA (25 (28.09%)/42 (47.19%) (p=0.541)); immediate cause of CA (35 (39.33%)/40 (44.94%) (p=1.000)); attempted resuscitation (14 (15.73%)/22 (24.72%) (p=0.435)); initial condition (37 (41.57%)/39 (43.82%) (p=1.000)); initial rhythm (42 (47.19%)/35 (39.33%) (p=1.000)); time of tracheal intubation (44 (49.44%)/62 (69.66%) (p=0.699)); CA provider (1 (1.12%)/1 (1.12%) (p=1.000)); and person responsible for the record (76 (85.39%)/74 (83.15%) (p=0.493)).

Concerning legibility assessment, all records proved to be legible and easy to read. Regarding the presence of erasures in nursing records, there were erasures in 11 (12.36%)/17 (19.10%) (p=0.153) at the preand post-intervention stages, respectively.

## DISCUSSION

The importance of adequate documentation in patients’ medical records is evident. Inadequate nursing records compromise care quality and safety, especially in situations of CA and CPR^(3,8)^. The quality of CA documentation is crucial to ensuring safe care, being fundamental in the care, management, ethical, and legal dimensions of healthcare practice. The lack of visible records can hinder audits and communication among the multidisciplinary staff, in addition to impacting hospital costs due to claim denials^(7)^.

According to the Code of Ethics for Nursing Professionals, it is the responsibility of professionals to encourage and ensure complete records in patients’ medical chart^(14)^. Weaknesses were observed in the completeness of recorded items, although compliance increased from 68.66% to 76.54% (p=0.001) after the intervention.

Incomplete documentation may be a result of stress generated by critical events such as CA^(15)^. The In-hospital Utstein Style guidelines, analyzed since the 1990s, aim to standardize research on in-hospital resuscitation, considering hospital, patient, and event variables^(5,11)^. Despite the manuals provided by the Federal Council of Nursing and the Regional Council of Nursing, the specific approach to CA records needs to be reviewed, creating protocols to address emergencies more effectively^(6,14)^.

In relation to date and time, the absence of a date was identified in six (6.74%) records before implementation and in one (1.12%) after. This lack compromises the validity of records and the ability to track the care provided. Although 95.51% of records had the time of event filled in, only 16.85% were complete, evidencing a persistent problem in temporal documentation^(16)^. Dated and legible records are essential to guarantee the authenticity of nursing documents^(6,14)^.

Critical information during CA includes initial rhythm, interventions performed, and time of event^(5)^. However, a reduction was observed in the completeness of initial CA rhythm recording, falling from 42 (47.19%) to 35 (39.33%) after the intervention. It is fundamental that professionals recognize the four cardiac rhythms of CA, as this classification directly affects the choice of treatment^(1)^. The ability to quickly identify CA and perform CPR is vital to improving survival rates^(17)^.

The variables related to patients’ initial condition and the immediate cause of CA showed low levels of agreement: 38 (42.70%) and 35 (39.33%), respectively. The absence of this information impacts patient assessment and prognosis. Patient comorbidities are rarely mentioned in the literature, despite their relevance to resuscitation outcomes^(5)^.

Following the intervention, resuscitation attempt documentation remained incomplete in 7.87% and only partially complete in 37.08% of records, often being summarized in vague expressions. The absence of data in medical records can raise ethical dilemmas, especially in patients under palliative care^(18)^. In Brazil, the decision not to resuscitate must be documented, but the lack of legal support is concerning^(19)^.

Although data on tracheal intubation were present in 74.16% of records, only 49.44% were complete. Documentation of medications and defibrillation was recorded in only 55.06%, making it difficult to analyze response time intervals^(1)^. The responsibility for initial recording of CA falls on nurses, who must document it accurately and completely^(17,20)^.

The 2020 American Heart Association guideline reinforces the correlation between the quality of records and survival in CA, encouraging the creation of specific databases^(1)^. The lack of complete information in nursing records is a global problem, compromising the implementation of evidence-based practices^(3-5)^.

Furthermore, continuing education and critical training of the healthcare staff are essential to improve the quality of CA documentation^(21,22)^. Approaches such as microlearning offer effective methods for knowledge retention^(23)^. It is necessary for institutions to implement continuous training, based on the reality of daily work^(24)^. Moreover, the initial training of professionals often fails to adequately prepare them for the job market^(17,25,26)^.

In light of the above, it is crucial to establish an organizational culture focused on quality, with the creation and monitoring of protocols that guarantee the effectiveness of health records. This study is innovative in assessing the quality of nursing records in CA before and after the intervention of the In-hospital Utstein Style protocol.

### Study limitations

Study limitations include the fact that it was a single-center study and that the pilot nursing documentation form was not implemented in all units of the hospital, being restricted to the AICU and AER, which may limit the generalizability of results, although it was conducted in high-complexity settings. Other aspects to consider are the fact that the form was not officially approved by the institution and that data collection was carried out during the SARS-CoV-2 pandemic, a fact that led the AER and AICU to adopt changes in care to ensure the safety of patients and professionals.

### Contributions to nursing, health, or public policy

This study contributed to demonstrating the relevance of compliance with nursing records in CA in daily practice, in addition to supporting the discussion on the need for the use of protocols, term standardization, and data uniformity. It is evident that continuous nursing training programs promote the quality of care provided and, consequently, patient safety.

## CONCLUSIONS

The overall compliance in completing the records showed an increase in the mean percentage of completion and completeness, respectively, when comparing the preand post-intervention periods of the In-hospital Utstein Style protocol as a support instrument for CA records in the AICU and AER, thus confirming the plausibility of the hypotheses of this research.

The importance of developing quality tools for nursing records in CA is evident due to their particularities in the routine of emergency care. Further studies are recommended to compare and monitor the evolution of CA care records.

## Data Availability

The research data are available only upon request.
