# Supplementary material for: Quality of nursing records in cardiac arrest based on the In-hospital Utstein Style protocol
Source: Rev Bras Enferm. 2026 Aug 3;79:e20240572. doi: 10.1590/0034-7167-2024-0572 (PMC13435857; doi:10.1590/0034-7167-2024-0572)
Supplement: Translation

# Translation: Qualidade dos registros de enfermagem em parada cardiorrespiratória baseada no protocolo *In-hospital Utstein Style*

**DOI:** 10.1590/0034-7167-2024-0572pt

**Authors:** Dhieizom Rodrigo de Souza, Lucia Tobase, Débora Rodrigues Vaz, Thatiane Facholi Polastri, Sandra Helena Cardoso, Heloisa Helena Cueto Peres

**Affiliations:** I Universidade de São Paulo. São Paulo, São Paulo, Brasil; II Centro Universitário São Camilo. São Paulo, São Paulo, Brasil

**Keywords:** Registros de Enfermagem, Cuidados de Enfermagem, Parada Cardíaca, Segurança do Paciente, Enfermagem.

## Abstract

**Objetivos:**

avaliar a qualidade dos registros de enfermagem em parada cardiorrespiratória baseados no *In-hospital Utstein Style*.

**Métodos:**

estudo quantitativo, quase-experimental, não randomizado, tipo antes-depois, com intervenção de impresso de apoio ao registro de enfermagem em parada cardiorrespiratória.

**Resultados:**

avaliaram-se conformidade geral para preenchimento e completude de 89 registros utilizando formulário de coleta elaborado a partir das variáveis do *In-hospital Utstein Style.* A conformidade geral dos registros de enfermagem apresentou um aumento na porcentagem média de preenchimento, passando de 55,57% para 65,25% (p=0,003), e na completude, de 68,66% para 76,54% (p=0,001), comparando os períodos pré e pós-intervenção.

**Conclusões:**

a intervenção apresentou efeito estatisticamente significativo nos índices de conformidade geral para preenchimento e completude dos registros de enfermagem em parada cardiorrespiratória. O estudo contribuiu para fomentar a importância do uso de protocolos como elemento essencial no registro de atendimento em parada cardiorrespiratória e na implementação de capacitação contínua da equipe de enfermagem.

## INTRODUÇÃO

A epidemiologia da parada cardiorrespiratória (PCR) intra-hospitalar ainda é limitada pela escassez de dados globais e regionais, especialmente em países de baixa e média renda^(1)^. Para melhorar a assistência e avaliar a eficácia na sobrevida pós-PCR, surgiram guias e protocolos específicos para o registro do evento^(2)^.

Os protocolos *Utstein Style* e *In-hospital Utstein Style*, desenvolvidos na década de 1990, oferecem diretrizes padronizadas para documentação de PCR extra-hospitalar e intra-hospitalar, respectivamente^(2)^. Esses registros permitem comparar o perfil epidemiológico da PCR entre diferentes serviços e países, organizando informações desde o momento da PCR até os desfechos, com acompanhamento do paciente até um ano após a alta^(3-5)^.

No Brasil, o *In-hospital Utstein Style* foi traduzido, adaptado e atualizado para aprimorar a coleta de dados, e equilibrar a padronização com a prática clínica e os desfechos assistenciais^(4)^. A enfermagem tem papel essencial na elaboração de registros, que não são apenas um dever legal, mas também uma ferramenta crucial para auditorias, ensino e pesquisas, promovendo o avanço científico e a melhoria contínua da assistência^(2,6,7)^.

Entretanto, a falta de informações completas durante o cuidado prejudica a avaliação da qualidade da reanimação e o desenvolvimento de indicadores assistenciais^(7)^. Como parte da equipe multiprofissional, a enfermagem deve garantir registros seguros e precisos, alinhados às metas de segurança e ao Programa Nacional de Segurança do Paciente^(8-11)^.

Embora estudos tenham avaliado a qualidade dos registros de enfermagem, poucos se dedicam especificamente à conformidade das anotações relacionadas à PCR^(2,3,11)^. Neste estudo, a conformidade foi avaliada considerando a completude dos itens do *In-hospital Utstein Style* nas anotações de PCR realizadas pela enfermagem. A ausência de um ou mais elementos padronizados foi entendida como não conformidade^(12)^.

Investigar a qualidade desses registros é essencial para fortalecer a segurança assistencial e garantir uma prática fundamentada em princípios éticos e legais. A hipótese do estudo é que, após intervenção educativa, os registros de enfermagem em PCR apresentam maior conformidade quanto ao preenchimento e à completude.

### Relevância do estudo

Este artigo é derivado da Dissertação nº 003219693^(13)^, depositada no Repositório Institucional da Universidade de São Paulo, disponível em: https://doi.org/10.11606/D.7.2022.tde-30102024-172947.

## OBJETIVOS

Avaliar a qualidade dos registros de enfermagem em PCR baseados no *In-hospital Utstein Style.*

## MÉTODOS

### Aspectos éticos

A pesquisa foi aprovada sob o parecer ético segundo a Resolução nº 466/2012 do Conselho Nacional de Saúde. O estudo foi conduzido de acordo com as diretrizes de ética nacionais e internacionais, e aprovado pelo Comitê de Ética em Pesquisa da Escola de Enfermagem da Universidade de São Paulo. O Termo de Consentimento Livre e Esclarecido foi obtido de todos os indivíduos envolvidos no estudo.

### Desenho, período e local do estudo

Trata-se de estudo quantitativo, quase-experimental, do tipo antes-depois, realizado entre janeiro e julho de 2021. O local do estudo foi um hospital universitário em São Paulo, com coleta de dados na Unidade de Terapia Intensiva Adulto (UTIA) e no Pronto-Socorro Adulto (PSA), devido à gravidade dos pacientes e maior ocorrência de PCR. A metodologia do estudo foi norteada de acordo com a diretriz *Standardsfor Quality Improvement Reporting Excellence* da plataforma *Equator Network.*

### Amostra e critérios de inclusão

A amostra do estudo foi constituída por 89 registros de PCR, coletados em cada fase da pesquisa. O cálculo amostral, baseado em 20 registros iniciais, determinou um tamanho de amostra capaz de detectar uma diferença de 55% na taxa de conformidade com um poder estatístico de 80%. Foram incluídos no estudo prontuários de pacientes que apresentaram PCR durante a última internação e que continham anotações completas e legíveis sobre o evento.

### Coleta de dados

Antes da intervenção, os dados foram coletados, retrospectivamente, em prontuários de pacientes que sofreram PCR entre julho de 2020 e fevereiro de 2021, sempre em ambiente privativo, para garantir o sigilo. Apenas registros de enfermagem foram analisados, com foco nas anotações e evolução de enfermagem. O formulário de coleta foi desenvolvido com base no protocolo *In-hospital Utstein Style*, contendo 14 variáveis agrupadas em três domínios:

- Pré-evento (dois itens): monitorização e causa imediata.
- Processo de PCR (seis itens): data e hora do evento, testemunha presente, intervenção de Suporte Avançado de Vida Cardiovascular (SAVC), ritmo inicial, tentativa de ressuscitação cardiopulmonar (RCP) e tratamentos durante o evento.
- Resultados (um item): desfecho do evento no hospital.

Além disso, foram incluídas variáveis suplementares, como condição clínica inicial, horários dos eventos, necessidade de intubação, provedor da PCR e legibilidade das anotações.

### Intervenção e capacitação

A capacitação ocorreu entre 1 e 5 de fevereiro de 2021, com 132 profissionais de enfermagem (60 da UTIA e 72 do PSA), incluindo oito residentes. A capacitação abordou a importância do registro de PCR baseado no protocolo *In-hospital Utstein Style*, com foco na completude dos dados.

Para isso, foi utilizado um *microlearning* de três minutos, com vídeo interativo e avatares explicando conceitos básicos e éticos, narrado pelo pesquisador. Após o vídeo, o pesquisador demonstrou o preenchimento do formulário e respondeu dúvidas. Cada sessão de capacitação durou 15 minutos.

### Fase pós-intervenção

Essa fase ocorreu no primeiro semestre de 2021, e os dados foram coletados dos prontuários para comparar a qualidade dos registros após a capacitação.

### Tratamento dos dados

A análise dos dados foi realizada por meio de estatística descritiva para caracterização da amostra e inferencial para testar as hipóteses. Para comparar as variáveis numéricas, utilizou-se o teste não paramétrico de Wilcoxon-Mann-Whitney. Para as variáveis categóricas, foi adotado o teste qui-quadrado de Pearson. O nível de significância referido para todos os testes foi de 5%. A conformidade dos registros foi calculada como a razão entre o número de registros completos e o total de registros avaliados, conforme a Figura 1.

**Figura 1:** Fórmulas de índice de conformidade geral e índice de conformidade por item

## RESULTADOS

Foram analisados 89 registros de enfermagem em PCR antes e após a intervenção, e verificaram-se, respectivamente, 63 (70,79%)/67 (75,28%) registros no PSA e 26 (29,21%)/22 (24,72%) registros na UTIA.

Observou-se aumento na média da conformidade do preenchimento dos registros de enfermagem em PCR, quando comparados com o protocolo *In-hospital Utstein Style* nos momentos pré-intervenção (55,57%) e pós-intervenção (65,25%) (p=0,003) nas UTIAs e no PSA. As variáveis que obtiveram índices significativos de conformidade relativa ao preenchimento, nos momentos pré e pós-intervenção, são apresentadas na Tabela 1.

**Tabela 1:** Apresentação da conformidade e não conformidade relativa ao preenchimento de cada variável do registro de enfermagem de parada cardiorrespiratória nos momentos pré e pós-intervenção do protocolo *In-hospital Utstein Style*, São Paulo, São Paulo, Brasil, 2021

Variável	Conforme Não conforme	Momento				Valor de *p* ^†^
		Pré		Pós		
		N	%	N	(%)	
Monitorizado	Conforme	40	44,94	58	65,17	0,007
	Não conforme	49	55,06	31	34,83	
Intervenções de SAVC/PCR	Conforme	27	30,34	45	50,56	0,006
	Não conforme	62	69,66	44	49,44	
Horário da IOT	Conforme	48	53,93	66	74,16	0,005
	Não conforme	41	46,07	23	25,84	
Tratamento durante o evento	Conforme	30	33,71	49	55,06	0,004
	Não conforme	59	66,29	40	44,94	
Provedor da PCR	Conforme	17	19,10	37	41,57	0,001
	Não conforme	72	80,90	52	58,43	

*PCR - parada cardiorrespiratória; †Valor de p - teste qui-quadrado de Pearson; SAVC - suporte avançado de vida cardiovascular; IOT - intubação orotraqueal.*

Nas demais variáveis, não foi observada relevância estatística, apesar da diferenciação dos valores de conformidade em registros nos momentos pré e pós-intervenção como: data da PCR (83 (93,26%)/88 (98,88%) (p=0,055)); PCR testemunhada (55 (61,68%)/64 (71,91%) (p=0,153); causa imediata da PCR (35 (39,33%)/40 (44,94%) (p=0,449)); tentativa de ressuscitação (56 (62,92%)/62 (69,66%) (p=0,343)); condição inicial do paciente (38 (42,70%)/40 (44,94%) (p=0,763)); ritmo inicial na PCR (43 (48,31%)/35 (39,33%) (p=0,701)); horário do evento (85 (95,51%)/86 (96,63%) (p=0,701)); e resultado do evento (86 (96,63%)/85 (95,51%) (p=0,701)).

Constatou-se, ainda, aumento na média de completude das informações documentadas nos registros de enfermagem de, respectivamente, 68,66% e 76,54% (p=0,001) antes e após a implementação do impresso de apoio nas UTIAs e no PSA.

As variáveis que obtiveram taxas de conformidade significativas em relação à completude, nos períodos pré e pós-intervenção, são apresentadas na Tabela 2.

**Tabela 2:** Distribuição da completude relativa ao preenchimento segundo variável do registro de enfermagem de parada cardiorrespiratória nos momentos pré e pós-intervenção do protocolo piloto do *In-hospital Utstein Style*, São Paulo, São Paulo, Brasil, 2021

Variável	Grau de completude	Momento				Valor de *p* ^†^
		Pré		Pós		
		N	%	N	(%)	
Horário do evento	Completo	15	16,85	27	30,34	0,020
	Parcialmente completo	34	38,20	19	21,35	
	Incompleto	36	40,45	40	44,94	
	Não se aplica	4	4,49	3	3,37	
Tratamento durante o evento	Completo	25	28,09	47	52,81	0,012
	Parcialmente completo	2	2,25	1	1,12	
	Incompleto	3	3,37	1	1,12	
	Não se aplica	59	66,29	40	44,94	
Resultado do evento	Completo	50	56,18	78	87,64	<0,001
	Parcialmente completo	30	33,71	2	2,25	
	Incompleto	6	6,74	5	5,62	
	Não se aplica	3	3,37	4	4,49	

*†Valor de p - teste qui-quadrado de Pearson.*

Nas demais variáveis, não foi observada relevância estatística nos resultados, apesar de apresentarem diferenciação dos valores de completude em registros nos momentos pré e pós-intervenção como: data (71 (79,78%)/75 (84,27%) (p=0,498)); testemunhado (52 (58,43%)/61 (68,54%) (p=0,712)); monitorizado (36 (40,45%)/54 (60,67%) (p=0,144)); intervenções de suporte avançado no momento da PCR (25 (28,09%)/42 (47,19%) (p=0,541)); causa imediata da PCR (35 (39,33%)/40 (44,94%) (p=1,000)); tentativa de ressuscitação (14 (15,73%)/22 (24,72%) (p=0,435)); condição inicial (37 (41,57%)/39 (43,82%) ((p=1,000); ritmo inicial (42 (47,19%)/35 (39,33%) (p=1,000)); horário de intubação traqueal (44 (49,44%)/62 (69,66%) (p=0,699)); provedor da PCR (1 (1,12%)/1 (1,12%) (p=1,000)); e responsável pelo registro (76 (85,39%)/74 (83,15%) (p=0,493)).

Quanto à avaliação de legibilidade, todos os registros se mostraram legíveis e de fácil leitura. Em relação à presença de rasuras nos registros de enfermagem, havia rasuras em 11 (12,36%)/17 (19,10%) (p=0,153) no momento pré e pós-intervenção, respectivamente.

## DISCUSSÃO

É evidente a importância de registros adequados no prontuário do paciente. Registros de enfermagem inadequados comprometem a assistência e segurança do cuidado, especialmente em situações de PCR e RCP^(3,8)^. A qualidade das anotações em PCR é crucial para garantir assistência segura, sendo fundamental nas dimensões assistenciais, gerenciais, éticas e legais da prática de saúde. A falta de registros visíveis pode prejudicar as auditorias e a comunicação entre a equipe multiprofissional, além de impactar custos hospitalares devido a glosas^(7)^.

De acordo com o Código de Ética dos Profissionais de Enfermagem, é responsabilidade dos profissionais incentivar e garantir registros completos no prontuário do paciente^(14)^. Observou-se fragilidade na completude dos itens registrados, ainda que a conformidade tenha aumentado de 68,66% para 76,54% (p=0,001) após a intervenção.

A documentação incompleta pode ser resultado do estresse gerado por eventos críticos como a PCR^(15)^. As diretrizes do *In-hospital Utstein Style*, analisadas desde os anos 90, visam padronizar pesquisas sobre reanimação hospitalar, considerando variáveis do hospital, paciente e evento^(5,11)^. Apesar de manuais dos sistemas Conselho Federal de Enfermagem e Conselho Regional de Enfermagem, a abordagem específica para registros de PCR deve ser revista, criando protocolos para atender a emergências de forma mais eficaz^(6,14)^.

Em relação à data e horário, identificou-se a ausência de data em seis (6,74%) registros, antes da implementação, e em um (1,12%), após. Essa falta compromete a validade dos registros e capacidade de acompanhar a assistência prestada. Embora 95,51% dos registros tivessem o horário do evento preenchido, apenas 16,85% estavam completos, evidenciando um problema persistente na documentação temporal^(16)^. Registros datados e legíveis são essenciais para garantir a autenticidade dos documentos de enfermagem^(6,14)^.

As informações críticas durante a PCR incluem ritmo inicial, intervenções realizadas e tempo do evento^(5)^. Contudo, observou-se uma redução na completude do registro do ritmo inicial da PCR, caindo de 42 (47,19%) para 35 (39,33%) após a intervenção. É fundamental que os profissionais reconheçam os quatro ritmos cardíacos da PCR, pois essa classificação afeta diretamente a escolha do tratamento^(1)^. A capacidade de identificar rapidamente a PCR e realizar a RCP é vital para melhorar as taxas de sobrevivência^(17)^.

As variáveis de condição inicial do paciente e causa imediata da PCR apresentaram conformidades baixas: 38 (42,70%) e 35 (39,33%), respectivamente. A ausência dessas informações impacta a avaliação e o prognóstico do paciente. Comorbidades dos pacientes raramente são mencionadas na literatura, apesar de sua relevância nos resultados da reanimação^(5)^.

Após a intervenção, a documentação da tentativa de reanimação permaneceu incompleta em 7,87% e apenas parcialmente completa em 37,08% dos registros, sendo frequentemente resumida a expressões vagas. A ausência de dados em prontuários pode levantar dilemas éticos, especialmente em pacientes sob cuidados paliativos^(18)^. No Brasil, a decisão de não reanimar deve ser documentada, mas a falta de respaldo legal é preocupante^(19)^.

Embora os dados sobre intubação traqueal estivessem presentes em 74,16% dos registros, apenas 49,44% estavam completos. A documentação de medicamentos e desfibrilação foi registrada em apenas 55,06%, dificultando a análise dos intervalos de tempo de atendimento^(1)^. A responsabilidade pelo registro inicial da PCR recai sobre o enfermeiro, que deve documentá-lo de forma fidedigna e completa^(17,20)^.

A diretriz da *American Heart Association* de 2020 reforça a correlação entre a qualidade dos registros e a sobrevivência em PCR, incentivando a criação de bancos de dados específicos^(1)^. A falta de informações completas nos registros de enfermagem é um problema global, comprometendo a implementação de práticas baseadas em evidências^(3-5)^.

Além disso, a educação permanente e formação crítica da equipe de saúde são essenciais para aprimorar a qualidade do registro da PCR^(21,22)^. Abordagens como *microlearning* oferecem métodos eficazes para retenção de conhecimento^(23)^. É necessário que as instituições implementem capacitação contínua, baseando-se na realidade do trabalho diário^(24)^. Além disso, a formação inicial dos profissionais muitas vezes falha em prepará-los adequadamente para o mercado de trabalho^(17,25,26)^.

Diante do exposto, é crucial estabelecer uma cultura organizacional voltada à qualidade, com a criação e monitoramento de protocolos que garantam a eficácia dos registros em saúde. Este estudo se mostra inovador ao avaliar a qualidade dos registros de enfermagem em PCR antes e após a intervenção do protocolo *In-hospital Utstein Style*.

### Limitações do estudo

Destacam-se, como limitações do estudo, o fato de se tratar de pesquisa unicêntrica e de o impresso piloto de apoio ao registro de enfermagem não ter sido implantado em todas as unidades do hospital, restringindo-se à UTIA e ao PSA, o que pode limitar a generalização dos resultados, embora tenha sido feito em contextos de alta complexidade. Outros aspectos a serem considerados são o fato de o impresso não ter sido oficializado pela instituição e de a coleta ter sido realizada durante o período da pandemia de SARS-CoV-2, fato que levou o PSA e a UTIA a adotar mudanças no atendimento para garantir a segurança dos pacientes e dos profissionais.

### Contribuições para as áreas da enfermagem, saúde ou políticas públicas

O presente estudo contribuiu para demonstrar a relevância da conformidade dos registros de enfermagem em PCR na prática diária, além de sustentar a discussão sobre a necessidade de utilização de protocolos, padronização de termos e uniformidade dos dados. Evidencia-se que programas contínuos de capacitação em enfermagem favorecem a qualidade da assistência prestada e, consequentemente, a segurança do paciente.

## CONCLUSÕES

A conformidade geral do preenchimento dos registros obteve aumento na média percentual de preenchimento e completude, respectivamente, na comparação dos momentos pré e pós-intervenção do protocolo *In-hospital Utstein Style* como instrumento de apoio dos registros de PCR na UTIA e no PSA, sendo possível confirmar a plausibilidade das hipóteses desta pesquisa.

Evidencia-se a importância do desenvolvimento de ferramentas de qualidade do registro de enfermagem em PCR por suas particularidades na rotina de atendimentos emergenciais. Recomenda-se a realização de outros estudos para comparar e acompanhar a evolução dos registros do atendimento de PCR.

## Data Availability

Os dados de pesquisa só estão disponíveis mediante solicitação.
